# Kinematic, kinetic and electromyographic response to customized foot orthoses in patients with tibialis posterior tenosynovitis, pes plano valgus and rheumatoid arthritis

**DOI:** 10.1093/rheumatology/ket337

**Published:** 2013-10-03

**Authors:** Ruth Barn, Mhairi Brandon, Daniel Rafferty, Roger D. Sturrock, Martijn Steultjens, Deborah E. Turner, James Woodburn

**Affiliations:** ^1^Institute for Applied Health Research, School of Health and Life Sciences, Glasgow Caledonian University, ^2^Centre for Rheumatic Diseases, Glasgow Royal Infirmary, Glasgow, UK.

**Keywords:** rheumatoid arthritis, foot orthoses, electromyography, kinematics, tibialis posterior

## Abstract

**Objective.** To describe the effect of customized foot orthoses (FOs) on the kinematic, kinetic and EMG features in patients with RA, tibialis posterior (TP) tenosynovitis and associated pes plano valgus.

**Methods.** Patients with RA and US-confirmed tenosynovitis of TP underwent gait analysis, including three-dimensional (3D) kinematics, kinetics, intramuscular EMG of TP and surface EMG of tibialis anterior, peroneus longus, soleus and medial gastrocnemius. Findings were compared between barefoot and shod with customized FO conditions.

**Results.** Ten patients with RA with a median (range) disease duration of 3 (1–18) years were recruited. Moderate levels of foot pain and foot-related impairment and disability were present with moderately active disease states. Altered timing of the soleus (*P* = 0.05) and medial gastrocnemius (*P* = 0.02) and increased magnitude of tibialis anterior (*P* = 0.03) were noted when barefoot was compared with shod with FO. Trends were noted for reduced TP activity in the contact period (*P* = 0.09), but this did not achieve statistical significance. Differences in foot motion characteristics were recorded for peak rearfoot eversion (*P* = 0.01), peak rearfoot plantarflexion (*P* < 0.001) and peak forefoot abduction (*P* = 0.02) in the shod with FOs compared with barefoot conditions. No differences in kinetic variables were recorded.

**Conclusion.** This study has demonstrated, for the first time, alterations in muscle activation profiles and foot motion characteristics in patients with RA, pes plano valgus and US-confirmed TP tenosynovitis in response to customized FOs. Complex adaptations were evident in this cohort and further work is required to determine whether these functional alterations lead to improvements in patient symptoms.

## Introduction

Pathology affecting the tibialis posterior (TP) tendon is common in RA and is frequently associated with a progressive flat foot deformity [pes plano valgus (PPV)] [1]. This condition has a negative impact on health-related quality of life and occurs in conjunction with moderate levels of foot-related impairment and disability [1, 2]. Both inflammatory and mechanical features have been shown to co-exist [2] and two studies have demonstrated abnormal foot motion combined with increased TP muscle activity in patients with RA and PPV [2, 3].

Treatment options are varied and the evidence base for interventions is limited. Typical treatments include foot orthoses (FOs) to reduce the mechanical strain on the TP tendon. There is evidence to suggest that FOs in RA reduce foot pain and plantar pressures, but questionable evidence for improving foot function [4]. While there is some evidence demonstrating that FOs improve rearfoot motion characteristics in PPV in RA [5], it is not known whether FOs re-establish normal function of TP in this patient group.

Only two studies have investigated the effect of FOs on EMG activity of TP during gait in participants with low-arched foot posture [6, 7] and only one of those combined EMG with kinematic data [6]. Stacoff *et al.* [6] reported no systematic changes in EMG activity of TP in five participants with four different orthoses conditions and high interindividual variation was present. Murley *et al.* [7] investigated the effect of two different types of FOs in 15 participants with low-arched foot posture and 15 with normal-arched foot posture. Both FOs significantly reduced TP EMG amplitude during gait compared with the shod-only condition, and additional changes were recorded with other lower limb muscles [7]. This preliminary evidence supports the theory of mechanical off-loading of the TP tendon; however, the study was undertaken in asymptomatic individuals. To advance our understanding, the next step was to determine whether these findings are replicated in symptomatic patient populations. Therefore the aim of this preliminary study was to investigate the effect of FOs on TP EMG in patients with RA, PPV and US-confirmed TP tenosynovitis and to combine this with detailed analysis of foot motion using a multisegmented foot model.

## Methods

### Patients

Patients were recruited from a consecutive sample at outpatient clinics in Glasgow Royal Infirmary and Gartnavel General Hospital, Glasgow, UK. Patients were eligible for inclusion if they had a confirmed diagnosis of RA based on the 1987 ACR criteria [8], passively correctable PPV deformity, US-confirmed TP tenosynovitis and had not received or worn FOs within the last 12 months. Tenosynovitis was confirmed clinically by the presence of tenderness and/or swelling along the course of the tendon and on US by the presence of hypoechoic or anechoic thickened tissue with or without fluid in the tendon sheath present in two planes and with or without power Doppler signal [9]. Ethical approval was obtained from the West of Scotland Local Research Ethics Committee (09/S0704/44) and NHS Greater Glasgow and Clyde Research and Development (GN09RH373). All participants provided informed, written consent in accordance with the Declaration of Helsinki.

### Demographic, disease and clinical assessment

Participant age, gender and disease duration were recorded and the most symptomatic limb was studied. A core set of clinical variables was recorded: tender and swollen foot joint counts undertaken by a single clinician (R.B.), foot posture using the Structural Index [10], foot-related impairment and disability using the Foot Impact Scale (FIS) for RA and global disability using the HAQ. Disease activity was recorded using 28-joint DAS (DAS28) with ESR within 2 weeks of assessment. Visual analogue scales (VASs, 100 mm) were used to record foot, general health and arthritis pain.

### Foot orthoses

All participants were provided with commercially manufactured customized FOs (Firefly Orthoses Ltd, Ireland) from a subtalar joint neutral cast. All prescriptions requested extrinsic rearfoot posting and intrinsic forefoot posting. All FOs were manufactured from polypropylene, with a 3-mm poron/vinyl covering to the toes and an additional 3-mm poron in the forefoot region for additional cushioning. All patients were given a minimum of a 10–15 min period of acclimatization prior to data collection.

### Biomechanical analysis

A 12-camera, 120-Hz, three-dimensional (3D) motion analysis system (Qualisys Oqus, Gothenburg, Sweden) was used to track the motion during gait of a multisegmented foot model comprising functional units for the shank, whole foot, rearfoot and forefoot (described in detail elsewhere [11]). A single force plate (Kistler, Winterthur, Switzerland) recorded ground reaction forces simultaneously. Data were collected in barefoot and shod with FO conditions using an adapted shoe (Flextop Diabetic Shoe, Reed Medical Ltd, UK) with windows cut to allow marker visualization during walking trials. In an attempt to lessen infection risk by reducing the time indwelling electrodes were *in situ* and to avoid patient fatigue, shod-only trials were not conducted. Visual 3D software (C-Motion, Inc., Rockville, MD, USA) was used to extract a core set of functional variables based on previous work and mapped to the foot deformity [2]: peak ankle joint moments and power, peak rearfoot eversion, rearfoot plantarflexion, forefoot abduction and forefoot dorsiflexion. Walking speed was self-selected and recorded using timing gates (Brower Timing Systems, Draper, UT, USA). Trials exceeding ±5% of the self-selected speed were excluded and a total of five walking trials were included for each participant.

### EMG analysis

In order to avoid undertaking an invasive procedure on participants at risk of infection, intramuscular EMG was restricted to the inaccessible TP muscle. TP EMG was undertaken using bipolar stainless steel nylon-coated fine wire electrodes (Motion Lab Systems Inc., Baton Rouge, LA, USA). Electrodes were inserted under US guidance (Esaote Mylab 70) using a 13–14 MHz linear array transducer via the posterior-medial approach at 50% of the distance between the medial malleolus and the tibial tubercle [12]. The accuracy of electrode placement was verified by checking the signal while applying manual resistance in the direction of dorsiflexion and eversion while participants were instructed to actively contract TP via plantarflexion and inversion. In addition, the signal was also checked when participants flexed their toes to ensure the electrode had not retracted into the flexor digitorum longus muscle. Tibialis anterior, soleus, peroneus longus (PL) and medial gastrocnemius EMG signals were recorded using Trigno (Delsys Inc., Boston, MA, USA) wireless surface electrodes applied following the Surface ElectroMyoGraphy for the Non-Invasive Assessment of Muscles (SENIAM) guidelines [13]. Surface electrodes had a single differential configuration, interelectrode distance of 10 mm, 4-bar formation, bandwidth of 20–450 Hz and 99.9% silver contact material. Discrete variables were recorded for each muscle relating to the peak of activity and the time of peak activity during contact and combined midstance/propulsive (MS/P) phases of stance based on when the muscles were most active [14]. Data were collected in barefoot and shod conditions within the same session due to the lack of reliability of EMG between time points [11, 15].

### Data processing

All EMG signals were high-pass filtered with a cut-off frequency of 20 Hz. All EMG data were subject to a root mean squared moving average of 25 ms. EMG data were normalized to maximum voluntary isometric contractions (MVICs); three MVICs were recorded for each muscle following the completion of walking trials. The MVIC data were recorded for 5 s with a gradual build-up of 2 s prior to maximal effort for the final 3 s. The peak value from a 0.5-s window obtained from the 3-s maximal effort of the MVIC was used as the reference value, similar to the methods reported elsewhere [14, 16]. All participants were verbally encouraged in a standard manner during the MVICs and a 1-min recovery period was set between repetitions. Kinematic data were subject to a fourth-order Butterworth low-pass filter with a cut-off of 6 Hz.

### Statistical analysis

Statistical analyses were performed using SPSS 17.0 (SPSS Inc., Chicago, IL, USA). Demographic and group characteristics were summarized as the mean (s.d.) or median (range). Biomechanical and EMG data were normalized to 100% of stance and conditions were compared using the paired Student’s *t*-test or Wilcoxon signed-rank test according to the distribution characteristics of the data.

## Results

### Group characteristics

Ten patients, six female and four male, with RA and US-confirmed TP tenosynovitis with a mean age (s.d.) of 50 (9) years and a median (range) disease duration of 3 (1–18) years were recruited (Table 1). Moderate levels of foot-related impairment and disability were recorded (Table 1).

**Table 1:** Demographic and disease characteristics

Variable	RA (*n* = 10)
Age, years	50 (9)
Gender (male:female)	4:6
Disease duration, median (range), years	3 (1–18)
Body mass index, kg/m^2^	30 (6)
DAS28 score	4.6 (1.6)
FIS_impairment subscale_, 0–21	14 (3)
FIS_disability subscale_, 0–30	21 (5)
HAQ	1.3 (0.6)
Foot pain VAS, 0–100 mm	46 (19)
General health VAS, 0–100 mm	44 (26)
Arthritis VAS, 0–100 mm	51 (19)
Structural Index, rearfoot, 0–7	2 (1)
Structural Index, forefoot, 0–12	4 (3)
Swollen foot joint count, 0–14	0 (1)
Tender foot joint count, 0–14	7 (3)
Barefoot walking speed, m/s	1.00 (0.14)
Weight-bearing rearfoot alignment, degrees	−7 (3)

Values are given as mean (s.d.) except where specified otherwise. By convention, eversion angles are expressed as negative.

### Kinematics and kinetics

**Fig. 1:**
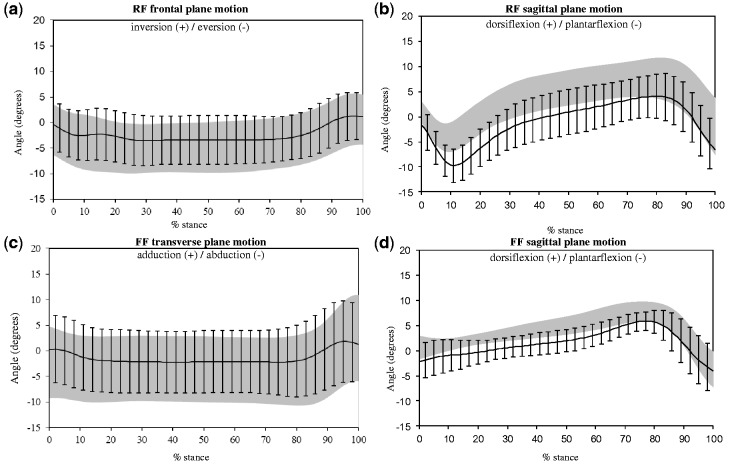
Motion time curves for key kinematic variables barefoot and shod with FO during stance. Data are presented as the mean (1 s.d.). Grey shaded area represents barefoot and black lines represent shod with FO. (**a**) Rearfoot frontal plane motion; (**b**) rearfoot sagittal plane motion; (**c**) forefoot transverse plane motion; (**d**) forefoot sagittal plane motion.

Alterations to the following variables were recorded: reduced peak rearfoot eversion, increased peak rearfoot plantarflexion and reduced peak forefoot abduction and dorsiflexion in the shod with FO condition compared to barefoot. Minimal differences to ankle joint moments and power were recorded. The findings are encouraging for the majority of discrete variables as the 95% CI of the difference did not cross zero and the significance level from a paired Student’s *t*-test was <0.05 (Table 2). The direction of the detected changes brings the values closer to those reported in the literature for control populations [2]. The motion time curves are presented in Fig. 1 for visual comparison of the conditions; moments and power are not presented due to the minimal discernible differences.

**Table 2:** Summary of kinematic and kinetic key discrete variables for barefoot and shod with FOs and the difference between the two conditions

Variable	Barefoot (*n* = 10)	Shod with FOs (*n* = 10)	Mean difference (95% CI)	Significance level
Peak RF eversion, degrees	−5 (5)	−4 (5)	−1 (−2, 0)	0.01*
Peak RF plantarflexion, degrees	−2 (6)	−7 (5)	5 (3, 7)	<0.001*
Ankle joint power, W/kg	1.7 (0.8)	1.6 (0.8)	0.05 (−0.03, 0.15)	0.17
Ankle joint moment, Nm/kg	−1.2 (0.3)	−1.2 (0.3)	0 00 (−0.00, 0.02)	0.26
Peak FF abduction, degrees	−5 (7)	−3 (7)	−1 (−2, 0)	0.02*
Peak FF dorsiflexion, degrees	8 (2)	7 (2)	2 (0, 4)	0.12

Data are presented as mean (s.d.) or mean difference (95% CI). Significance level is from paired samples *t*-tests. By convention, eversion, plantarflexion, abduction and ankle joint moments are expressed as negative values. Positive values for mean difference indicate the value was greater in the shod with FO condition. FF: forefoot; RF: rearfoot. *Significance level < 0.05.

### Electromyography

**Fig. 2:**
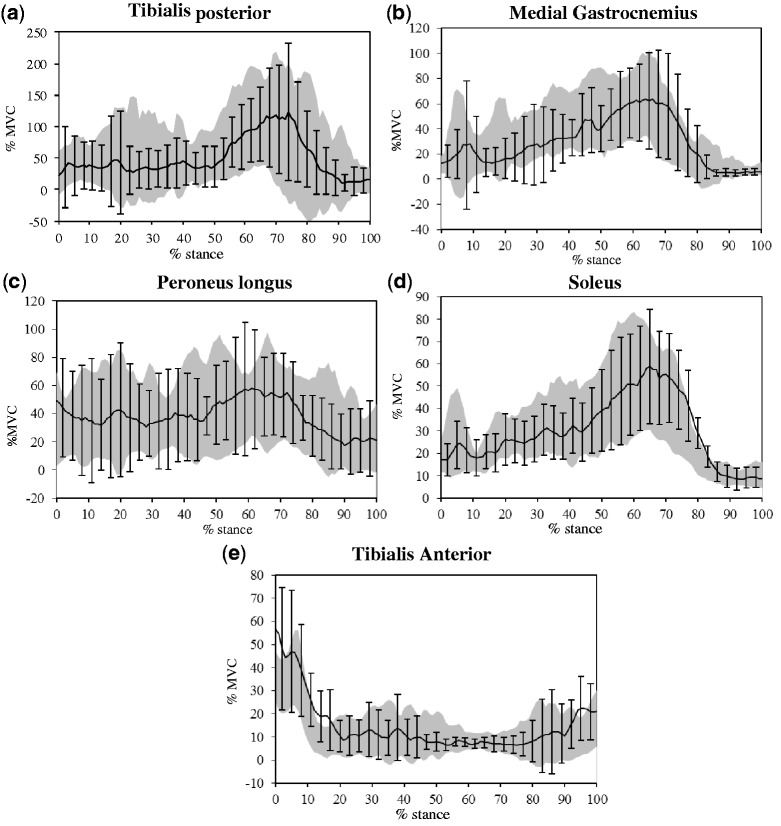
Ensemble EMG activity for lower limb muscles barefoot and shod with FO during stance. Barefoot represented by grey shaded area; black lines represent shod with FO. All data presented as mean (±1 s.d.). (**a**) Tibialis posterior; (**b**) medial gastrocnemius; (**c**) peroneus longus; (**d**) soleus; (**e**) tibialis anterior.

EMG data were not normally distributed, therefore the median [interquartile range (IQR)] values are presented in Table 3 along with the significance level from a Wilcoxon signed-rank test. In the majority of cases the IQR crossed zero, limiting the interpretation of results. However, the following variables demonstrated a difference between barefoot and shod with FO conditions confirmed by a significance level ≤0.05 and an IQR that did not cross zero: later peak of contraction of the gastrocnemius, later peak of contraction of the soleus and increased magnitude of tibialis anterior in the shod with FO condition compared with barefoot. The IQR of the TP peak in the contact phase did not cross zero, however, the significance value was 0.09, indicating a weak trend towards a reduction in the magnitude of contraction in shod with FO compared with barefoot. The ensemble averages of muscle activation profiles during stance are presented in Fig. 2 for visual comparison.

**Table 3:** Summary of EMG discrete variables for barefoot and shod with FO and the difference between the two conditions

Muscle	Variable	Barefoot (*n* = 10)	Shod + FO (*n* = 10)	Median of differences (IQR)	Significance level
Medial gastrocnemius	Peak MS/P	83 (59, 128)	89 (48, 129)	−7 (−14, 10)	0.47
	Time peak MS/P	46 (34, 65)	59 (43, 67)	5 (2, 14)	0.02*
PL	Peak contact	43 (28, 86)	46 (27, 58)	−2 (−10, 16)	0.87
	Time peak contact	9 (5, 15)	2 (0, 8)	−5 (−13, 1)	0.09
	Peak MS/P	70 (43, 105)	68 (56, 85)	4 (−13, 12)	0.68
	Time peak MS/P	68 (38, 77)	67 (58, 71)	−1 (−5, 21)	0.90
Soleus	Peak MS/P	69 (31, 84)	67 (39, 88)	4 (1, 8)	0.16
	Time peak MS/P	61 (48, 63)	66 (63, 72)	7 (3, 18)	0.05*
Tibialis anterior	Peak contact	49 (32, 56)	53 (33, 85)	8 (2, 27)	0.03*
	Time peak contact	6 (0, 6)	1 (0, 4)	−2 (−5, 0)	0.23
TP	Peak contact	48 (35, 117)	34 (15, 94)	−14 (−31, −6)	0.09
	Time peak contact	13 (8, 15)	12 (6, 15)	0 (−2, 1)	0.67
	Peak MS/P	94 (56, 261)	126 (57, 215)	2 (−62, 47)	1.0
	Time peak MS/P	64 (60, 68)	66 (60, 73)	1 (−5, 9)	0.67

MS/P: midstance/propulsive period of stance. Data presented as median (IQR). A positive value for the median difference indicates the magnitude is greater or the timing occurs later in the shod with FO condition compared with barefoot. Significance level from Wilcoxon signed-rank test. *Significance level ≤ 0.05.

## Discussion

The aim of this study was to investigate the effect of customized FOs on TP muscle activation and kinematic and kinetic features in patients with RA, US-confirmed TP tenosynovitis and PPV. The current study is the first to investigate the effect of FOs on EMG activity of TP in patients with RA and PPV. The response of the TP and lower limb muscles to the FOs was variable, but there was a trend towards reduced activity of TP in the contact period; however, this did not reach statistical significance. Key discrete kinematic variables were improved as a result of the FOs, with values moving closer to those observed in control populations [2]. Further work is required to determine whether these functional alterations lead to improvements in patient symptoms. The results of this study add new data to an important but under-researched area, however, the results must be considered within the context of moderate levels of foot-related impairment and disability and moderately active disease states.

Available literature has investigated the effect of FOs on different muscle groups in walking and running conditions, however, due to varied methodologies, cohorts, types of FOs and follow-up periods, data are unable to be pooled and evidence levels remain weak. Magnitude variables for tibialis anterior and gastrocnemius and soleus timing demonstrated encouraging results, but findings for TP fell short of statistical significance. These findings are contrary to those of Murley *et al.* [7], where a reduction in TP magnitude in the contact phase and an increase in the combined MS/P phase were reported when shod and shod with FO conditions were compared in a group of asymptomatic flat-footed participants. The reported changes were following an average of 12 days of wear for two types of FOs, i.e. on average, 6 days wear for each device [7]. An acclimatization period for FOs is usually deemed appropriate, in line with standard clinical practice. In the present study acclimatization was approximately 10–15 min, which may not be sufficient time to allow the neuromotor system to respond optimally to the FOs and alter muscular control. However, consistent immediate EMG effects in response to FOs have been reported elsewhere in the literature [17]. Realigning mechanics may not have an immediate effect on learned compensatory mechanisms and as such the relative ‘plasticity’ of the neuromotor system is not clear.

The devices were manufactured from polypropylene, which is a semi-rigid material that will alter the foot–shoe interface. These devices may not always be comfortable at first use. Moreover, the studied cohort had moderate levels of self-reported foot pain, which further emphasizes the need for an appropriate acclimatization period. Ideally the effect of FOs would be studied over time, but EMG has been shown to be unreliable between time points and caution should be exercised when attempting to derive intervention effects if the electrodes have been removed and replaced [11, 15]. Furthermore, while relatively few changes were reported in muscle activity in this study, only muscles below the knee were studied and FOs may have a more significant effect on more proximal muscles [18].

Despite the lack of significant results for alterations to lower limb muscle activation, significant results were found for key discrete kinematic variables. Much of the available literature pertaining to the effect of FOs on kinematics and kinetics are from control populations with normal foot posture where varied FOs and/or levels of wedging are applied during walking or running [19–23]. The results are therefore not transferable to patient populations with foot deformity. Only one study has employed 3D motion analysis to evaluate FOs in RA and demonstrated improvements in rearfoot motion characteristics [5]. Exploiting advances in technology, this study has provided detailed information on the intersegment kinematics of conceptually relevant joints to underlying impairments in this patient group and demonstrated improvements in both rear- and forefoot motion characteristics as a result of customized FOs.

No significant differences were recorded for moments and power; however, these variables were recorded for the sagittal plane only. FOs were prescribed in this cohort to correct postural abnormalities primarily affecting the frontal and transverse planes (i.e. rearfoot eversion and forefoot abduction), and due to the complexities of the protocol, detailed kinetic analysis in these planes and at small segments within the foot was not undertaken. It is anticipated that customized FOs will alter the external ground reaction force moment arm length and thereby reduce the associated internal moment and subsequent strain on the soft tissues. There was a statistically significant increase in peak rearfoot plantarflexion that was accompanied by an increase in tibialis anterior activity in the contact period, perhaps in an attempt to control the increased sagittal plane movement despite the non-significant result for sagittal plane moment. The reduction in rearfoot eversion was not accompanied by a reduction in TP activity in this cohort, which demonstrates the complexity and multifactorial nature of the deformity.

This study was subject to four main limitations. First, the complexities of the protocol resulted in a small sample being recruited and therefore it is difficult to draw robust conclusions from the data due to lack of power. However, encouraging preliminary observations were made that highlight the effects of FOs on foot motion in RA-associated PPV. Second, the patients in this study had moderate levels of foot pain and foot-related impairment and disability in conjunction with moderately active disease. It is likely these features affected the outcomes of the study and the global effects of the disease cannot be overlooked when undertaking detailed analysis of the lower limb. The patient symptoms may also have influenced the ability to undertake a maximal voluntary contraction and therefore potentially influenced the EMG results. Third, the analysis compared only barefoot and shod with FOs, due to the complexities of the protocol, which does not separate the effect of the footwear from the FOs. The aim of this study was to determine the effect of FOs, and FOs are administered in conjunction with footwear as standard practice, therefore the treatment effect of the shoe was beyond the scope of this study with an already detailed protocol. However, the footwear was standardized across the group as the participants’ own footwear could not be used to capture the kinematic data. Finally, while there is not an accepted standard in terms of acclimatization period for FOs, previous studies reporting significant differences in muscle activity between the barefoot and shod with FO conditions have included wear times ranging from 6 days to 4 weeks [7, 23–25]. Therefore the habituation period in the present study was sufficient with regard to initial comfort levels, but it is possible greater changes may have been detected with a longer acclimatization period. In addition to these limitations, the results must be considered in terms of the high levels of variation present within and between participants. Suggestions for future work include extending this approach to a large-scale intervention study, using kinematic data to identify potential therapeutic targets and optimizing FO design to provide targeted, personalized, early interventions.

In summary, this study has demonstrated for the first time changes in muscle activation profiles and kinematics in response to FOs in patients with RA, PPV and US-confirmed TP tenosynovitis. Despite a minimal acclimatization period and moderate levels of foot-related impairment and disability, differences were detected in muscle activity and kinematic profiles in the rearfoot and forefoot segments. PPV in RA is a complex and multifactorial deformity and further work is required to determine whether these functional alterations will lead to an improvement in patient symptoms over time.

**Rheumatology key messages**

- This is the first study to investigate orthoses in RA, pes plano valgus and tibialis posterior tenosynovitis.
- Foot motion and muscle activation characteristics are altered in response to customized foot orthoses.
